# Identification of Functional lncRNAs Associated With Ovarian Endometriosis Based on a ceRNA Network

**DOI:** 10.3389/fgene.2021.534054

**Published:** 2021-01-27

**Authors:** Jian Bai, Bo Wang, Tian Wang, Wu Ren

**Affiliations:** Department of Obstetrics and Gynecology, Tongji Hospital, Tongji Medical College, Huazhong University of Science and Technology, Wuhan, China

**Keywords:** ovarian endometriosis, lncRNA, ceRNA network, topological analysis, random walk with restart

## Abstract

**Background:**

Endometriosis is a common gynecological disease affecting women of reproductive age; however, the mechanisms underlying this condition are not fully clear. The aim of this study was to identify functional long non-coding RNAs (lncRNAs) associated with ovarian endometriosis for potential use as biomarkers and therapeutic targets.

**Methods:**

RNA-seq profiles of paired ectopic (EC) and eutopic (EU) endometrial samples from patients with ovarian endometriosis were downloaded from the publicly available Gene Expression Omnibus (GEO) database. Bioinformatics algorithms were used to construct a network of ovarian endometriosis-related competing endogenous RNAs (ceRNAs) and to detect functional lncRNAs.

**Results:**

A total of 4,213 mRNAs, 1,474 lncRNAs, and 221 miRNAs were identified as being differentially expressed between EC and EU samples, and an ovarian endometriosis-related ceRNA network was constructed through analysis of these differentially expressed RNAs. H19 and GS1-358P8.4 were identified as key ovarian endometriosis-related lncRNAs through topological feature analysis, and RP11-96D1.10 was identified using a random walk with restart algorithm.

**Conclusion:**

Based on bioinformatics analysis of a ceRNA network, we identified the lncRNAs H19, GS1-358P8.4, and RP11-96D1.10 as being strongly associated with ovarian endometriosis. These three lncRNAs hold potential as targets for medical therapy and as diagnostic biomarkers. Further studies are needed to elucidate the detailed biological function of these lncRNAs in the pathogenesis of endometriosis.

## Introduction

Endometriosis is a common benign gynecological disorder with no clear pathogenesis (Dunselman et al., 2014). Endometriotic tissue commonly presents as lesions in the pelvic cavity formed by implantation and conglutination at ectopic locations, which cause irregular menstruation, chronic pelvic pain, and infertility (Adamson, 2011; Johnson et al., 2017). Ovarian, peritoneal, and deep infiltrating endometriosis are the main three types of endometriosis (Kinkel et al., 2006). Overall, it is estimated to affect approximately 10% of women of reproductive age, increasing to 35–50% in symptomatic patients (Giudice and Kao, 2004; Bazot and Daraï, 2017). Although many theories have been put forth to explain the etiology of endometriosis, such as the retrograde menstruation theory and stem cell theory, the pathogenesis of endometriosis has not yet been clearly established (Sampson, 1927; Maruyama and Yoshimura, 2012). As the mechanism underlying the development of this disease remains obscure, it presents a challenge for early diagnosis and treatment, due to a lack of effective biological markers and treatment targets. It is generally believed that the occurrence and development of endometriosis is a complex biological process involving multiple genes and factors, so we must firstly detect those genes which have important functions in the process of endometriosis.

Long non-coding RNAs (lncRNAs) are a major class of ncRNAs of greater than 200 nucleotides in length, which can regulate gene activity at transcriptional, post-transcriptional, and epigenetic levels (Geisler and Coller, 2013; Xiong et al., 2016). And an increasing number of studies demonstrate that aberrantly expressed lncRNAs are associated with various diseases such as carcinoma (Du et al., 2013), neurodegenerative disease (Riva et al., 2016), and endometriosis (Panir et al., 2018). For example, lncRNA LINC00261 was reported to inhibit cell growth and migration in endometriotic tissue (Sha et al., 2017), and AC002454.1 was reported to alter cell cycle progression by regulating CDK6 and promoting the proliferation of endometriotic cells in the secretory phase, thus participating in the progression of endometriosis (Wang et al., 2015; Tigan et al., 2016). Another lncRNA, MALAT1, mediates hypoxia-induced autophagy and activates the ERK/MAPK signaling pathway through p21/p53-dependent cell cycle entry in endometriotic cells (Li et al., 2019). Theoretical and experimental studies have demonstrated that lncRNAs can compete with mRNAs for microRNA (miRNA) molecules to regulate mRNA expression, thus constituting a form of competing endogenous RNA (ceRNA) crosstalk (Salmena et al., 2011). These lncRNA-mRNA-miRNA regulatory associations have been observed in many diseases, including endometrial cancer; lncRNA H19, for example, was reported to accelerate tumor formation in endometrial cancer by regulating HIF-1α/AXL signaling through competition for miR-20b-5p (Zhu et al., 2019). Many studies have used ceRNA network construction and analysis to identify prognostic biomarkers for endometrial cancer (Wang et al., 2019; Xia et al., 2019; Zhao et al., 2019), but there have been few systematic studies of lncRNA-mRNA ceRNA networks in endometriosis.

In this study, we used RNA-seq data from the Gene Expression Omnibus (GEO) to integrate regulatory relationships among lncRNAs, miRNAs, and mRNAs, and identified lncRNA–mRNA competing interactions involved in ovarian endometriosis. We then constructed a ceRNA network for ovarian endometriosis and investigated functional lncRNAs with potential roles in the development of endometriosis.

## Materials and Methods

### mRNA- and miRNA-seq Profiles

mRNA-seq and miRNA-seq profiles (GSE105764 and GSE105765, respectively) based on the Illumina HiSeq 4000 platform were downloaded from the publicly available GEO database. These datasets were derived from eight paired ectopic (EC) and eutopic (EU) endometrial samples obtained from eight Chinese female patients with ovarian endometriosis.

### Acquisition of lncRNA Expression Profiles

LncRNA transcript sequences and protein-coding gene annotation profiles were downloaded from the GENCODE database^1^ (Harrow et al., 2012). We identified lncRNAs and protein-coding genes using their official symbol names and Ensembl IDs (GENCODE v.32).

### Expression Profile Analysis

mRNAs, lncRNAs, and miRNAs showing differential expression between EC and EU samples were identified using the empirical Bayesian method implemented in the R “limma” package (Ritchie et al., 2015). An adjusted *p*-value of < 0.05 with fold change >2 was considered statistically significant.

### MiRNA–mRNA and miRNA–lncRNA Interactions

MiRNA-mRNA interactions and miRNA-lncRNA interactions were obtained from the publicly available Starbase v.2.0 database^2^ (Li et al., 2014). Starbase v.2.0 database identified miRNA–mRNA and miRNA–lncRNA interactions from 107 CLIP-Seq (HITS-CLIP, PAR-CLIP, iCLIP, and CLASH) datasets. We got 423,975 miRNA-mRNA interaction pairs, which included 13,805 mRNAs and 386 miRNAs, and 10,212 miRNA-lncRNA interaction pairs, which included 1,127 lncRNAs and 277 miRNAs. Differentially expressed mRNAs, lncRNAs, and miRNAs were then mapped to the interactions for further selection.

### Hypergeometric Test

After combining miRNA–mRNA and miRNA–lncRNA interactions, a global triple network was constructed. Then, candidate lncRNA-mRNA ceRNA pair was selected using the hypergeometric test as follows:

$$P=1-\sum_{k=0}^{r-1}\frac{\begin{array}{c} \left(\begin{array}{c} t\\ k \end{array}\right)\,\left(\begin{array}{c} T-t\\ N-k \end{array}\right) \end{array}}{\left(\begin{array}{c} T\\ N \end{array}\right)}$$

where T is the total number of human miRNAs; t is the number of miRNAs interacting with mRNAs; N is the number of miRNAs interacting with lncRNAs; and r is the number of miRNAs shared by the mRNAs and lncRNAs. A *p*-value of < 0.01 was considered statistically significant.

### Network Generation and Topological Analysis

**FIGURE 1:**
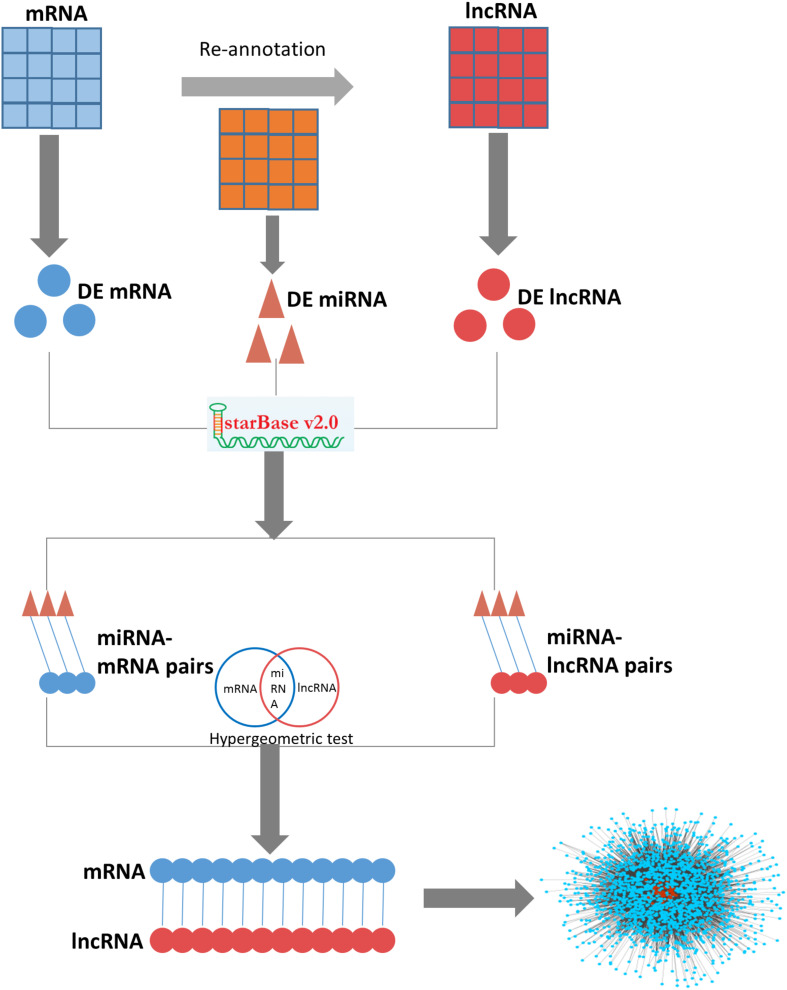
The pipeline for construction of the ovarian endometriosis-related ceRNA network.

The lncRNA-mRNA ceRNA network for ovarian endometriosis was constructed based on the “ceRNA hypothesis” (pipeline shown in Figure 1). To assess network characteristics, we computed degree centrality, betweenness centrality, and closeness centrality of each node in the network, and analyzed their distribution. Degree is the most elementary feature in networks, and it is defined as the number of edges linked to a node. If the degree distribution of a given network follows a power law, the network will have only a few nodes with a large number of edges (i.e., hubs). Betweenness is a centrality which counts the number of shortest paths that pass a node, and it represents close connectivity of a node in the network. Closeness centrality measures how close one node is to all other nodes in the network, and higher values indicate higher centrality. We calculated the top ten nodes, ranked according to degree, betweenness, and closeness features.

### Random Walk With Restart on Ovarian Endometriosis Related ceRNA Network

To prioritize ovarian endometriosis-related lncRNAs, we performed Random walk with restart (RWR) algorithm analyses on the ceRNA network using R “RandomWalkRestartMH” package (Köhler et al., 2008; Le and Kwon, 2013). This method simulates a walker that randomly moves from the current node to neighboring nodes with a probability (r) in the walk, and is described as follows:

$${\text{P}}^{t+1}=\left(1-\text{r}\right)\,{\text{WP}}^{\text{t}}+{\text{rP}}^{0}$$

where P^*t*^ is a vector in which the ith element holds the probability of being at node i at time step t; P^0^ is the initial probability vector; W is the column-normalized adjacency matrix of the network; and r is the restart probability of the random walk at every step. When the difference between P^*t*^ and P^*t*+1^ fell below a given cutoff value, the update procedure was terminated, and P^*t*+1^ was the output value of the RWR algorithm.

We obtained a list of ovarian endometriosis-related genes from DisGeNET^3^ and selected four genes (ESR1, SNCG, PTCH1, and MUC1) which were also included in the network to use as seeds in the RWR model (Piñero et al., 2017). The initial probability, P^0^, of each seed mRNA was set as 1/n (where n is the number of seed mRNAs), while other non-seed mRNAs were set as 0. The *r* value was set to 0.5. The stable probability P^∞^ of each non-seed mRNA can be obtained when the difference between P^*t*^ and P^*t*+1^ is less than 10^–10^. All candidate lncRNAs were sorted based on P^∞^, and lncRNAs with high scores were considered the most likely to be disease-related. Statistical significance was determined by comparing the scores of lncRNAs in the network following 10,000 iterations with shuffling of the known endometriosis-related genes. While M was the time that the score of a lncRNA was higher than the real one and N was the total iterating time, a *p*-value for the lncRNA was calculated by the ratio of M and N. A *p*-value < 0.01 was considered statistically significant.

### Functional Evaluation

Functional evaluation of lncRNAs was performed by calculating the Pearson correlation coefficient (PCC) between mRNAs and lncRNAs, and targeted mRNAs with *p*-value < 0.05 and PCC > 0 were used to implement enrichment analysis on Database for Annotation, Visualization, and Integrated Discovery (DAVID v.6.8) (Huang et al., 2009).

## Results

### Differentially Expressed RNAs

**FIGURE 2:**
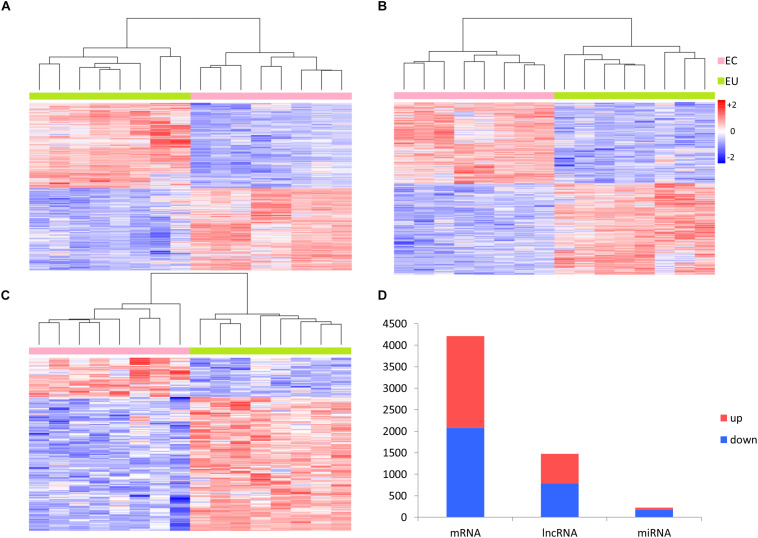
Differential expression analysis of RNAs. Heat-map generated by unsupervised clustering shows differentially expressed **(A)** mRNAs, **(B)** lncRNAs, and **(C)** miRNAs. **(D)** Distribution of upregulated and downregulated RNAs in ectopic endometrial samples vs. eutopic endometrial samples. EC, ectopic endometrial samples; EU, eutopic endometrial samples; Up, upregulated; Down, downregulated.

A total of 19,697 mRNAs, 15,473 lncRNAs, and 1,556 miRNAs were selected from annotation profiles for further study. We compared the expression profiles of mRNAs, lncRNAs, and miRNAs in paired EC and EU samples using the R “limma” package, and 4,213 mRNAs, 1,474 lncRNAs, and 221 miRNAs were identified as being differentially expressed (adjusted *p*-value < 0.05, fold change >2), which can be used to separate the EC and EU samples (Figure 2).

### Construction of the ceRNA Network

**FIGURE 3:**
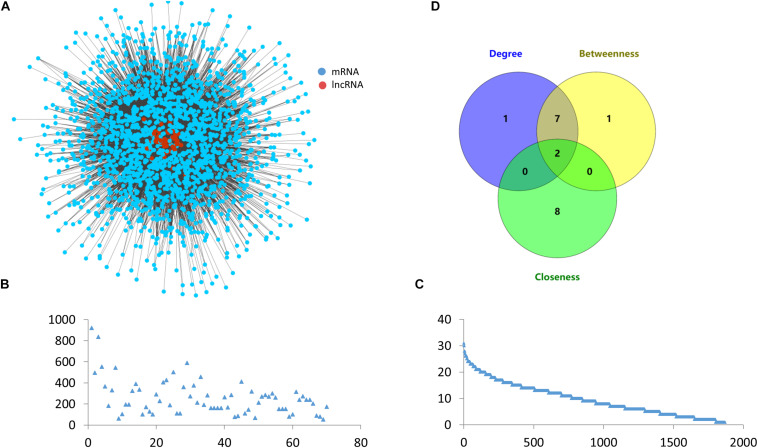
Topological features of the ceRNA network. **(A)** Global view of the ovarian endometriosis-related lncRNA-mRNA ceRNA network. **(B)** Degree distribution of lncRNAs in the ceRNA network. **(C)** Degree distribution of mRNAs in the ceRNA network. **(D)** Venn diagram showing the overlap of the top ten nodes in topological features of degree, betweenness, and closeness.

The ovarian endometriosis-related ceRNA network was constructed based on the ceRNA hypothesis by integrating expression profile data with miRNA-mRNAs and miRNA-lncRNA regulatory relationships. As described in the “Materials and Methods” section, a lncRNA–mRNA competing interaction pair was selected if the lncRNA and the mRNA significantly shared common miRNAs and the miRNAs were also differentially expressed. As a result, we identified 18,064 dysregulated lncRNA-mRNA pairs associated with ovarian endometriosis after the hypergeometric test, including 1,869 mRNAs and 70 lncRNAs (Figure 3A).

### Topological Analysis of Ovarian Endometriosis Related ceRNA Network

Degree, betweenness, and closeness are the three most important topological features in network analysis. We therefore analyzed these common topological measurements to reveal the characteristics of the ceRNA network. We also draw the degree distributions of mRNAs and lncRNAs (Figures 3B,C). As we can see these two distributions all follow a similar power law, which shows the stability of the ceRNA network. We also ranked the top ten nodes for each of the topological features (Table 1), and found that two nodes, H19 and GS1-358P8.4, were in all three of the lists (Figure 3D). These two lncRNAs can therefore be regarded as hubs in the network.

**TABLE 1:** Top ten nodes in topological analyses of degree, betweenness, and closeness.

**Degree**	**Betweenness**	**Closeness**
H19	H19	H19
GS1-358P8.4	GS1-358P8.4	RECK
RP3-523K23.2	RP3-523K23.2	ITGB8
AC016747.3	RP11-96D1.10	JAZF1
DLX6-AS1	DLX6-AS1	GS1-358P8.4
RP11-96D1.10	AC016747.3	DOCK4
HOXA-AS2	RP11-284N8.3	CDC42SE2
RP11-54O7.1	RP11-379K17.4	TET3
HOXA-AS4	HOXA-AS2	TNFAIP3
RP11-284N8.3	RP11-54O7.1	PHTF2

### Function Annotation of Hub lncRNAs

**FIGURE 4:**
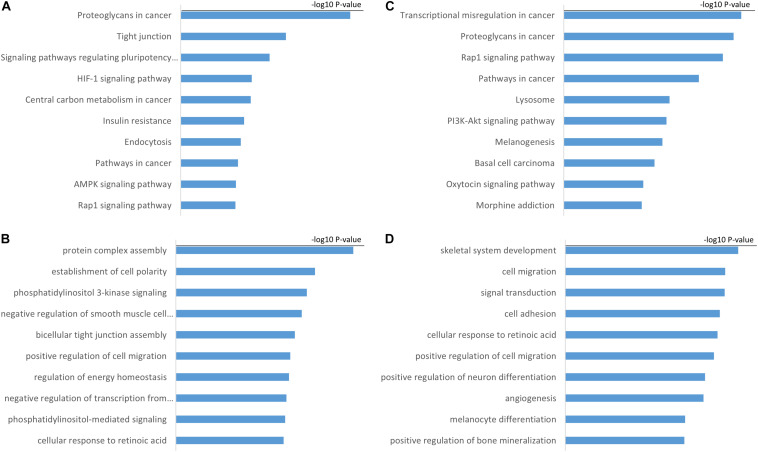
Functional annotation of hub lncRNAs. **(A)** KEGG pathway and **(B)** GO biological process analyses of H19. **(C)** KEGG pathway and **(D)** GO biological process analyses of GS1-358P8.4.

We performed Pearson correlation analysis between H19, GS1-358P8.4, and their targeted mRNAs, and selected mRNAs with a *p*-value < 0.05 and PCC >0. Gene ontology (GO) and the Kyoto Encyclopedia of Genes and Genomes (KEGG) analyses were then performed using these targeted mRNAs to assess the function of hub lncRNAs (Figure 4).

Our analyses showed that H19 is mainly associated with KEGG pathway of proteoglycans in cancer, tight junctions, and signaling pathways regulating the pluripotency of stem cells. Tight junctions are protein networks that control multiple cell functions such as junctional dynamics, cell migration, and proliferation (Balda and Matter, 2016). H19 is also associated with GO biological process of bicellular tight junction assembly and positive regulation of cell migration, which is consistent with the results of KEGG pathway analysis. GS1-358P8.4 is mainly associated with transcriptional misregulation in cancer, proteoglycans in cancer, and the Rap1 signaling pathway. Transcriptional misregulation is characteristic of endometriotic cells as well as cancer cells.

### Random Walk With Restart Analyses of the ceRNA Network

**FIGURE 5:**
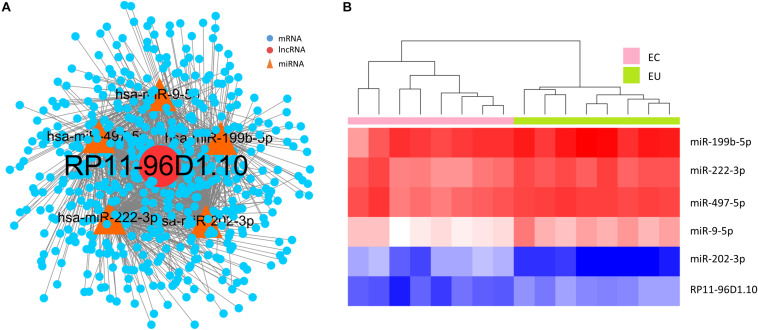
RP11-96D1.10 and its interacting miRNAs. **(A)** The triple lncRNA-miRNA-mRNA network of RP11-96D1.10. **(B)** Unsupervised clustering of RP11-96D1.10 and its interacting miRNAs can be used to discriminate between EC and EU samples. EC, ectopic endometrial samples; EU, eutopic endometrial samples.

We performed RWR algorithm analyses of the ceRNA network using four validated ovarian endometriosis-related genes (ESR1, SNCG, PTCH1, and MUC1) as seed nodes, prioritizing ovarian endometriosis-related lncRNAs, and we also performed permutation tests. After 10,000 iterations with permutation, we identified only one lncRNA, RP11-96D1.10, with *p* < 0.01. As the same lncRNA was also identified as a hub node in the network based on degree and betweenness analyses, RP11-96D1.10 may play an important role in the development of ovarian endometriosis. We therefore constructed a sub-network based on the lncRNA-miRNA-mRNA model for RP11-96D1.10 (Figure 5A). A total of five miRNAs interact with RP11-96D1.10, and its role was assessed by examining these miRNAs (Table 2). Of the five miRNAs, miR-202-3p was found to be upregulated in EC endometrial tissue, and Zhang et al. reported that this miRNA promotes the proliferation and invasion of endometriotic cells (Hawkins et al., 2011; Zhang et al., 2015). Furthermore, we were able to use RP11-96D1.10 and its interacting miRNAs to discriminate between EC and EU samples (Figure 5B).

**TABLE 2:** Functional lncRNAs and their interacting miRNAs.

**lncRNA**	**Expression**	**Location**	**miRNA**
H19	Down	Chromosome 11: 1,995,176-2,001,470	miR-106a-5p miR-106b-5p miR-107
			miR-130b-3p miR-138-5p miR-17-5p
			miR-18a-5p miR-18b-5p miR-196b-5p
			miR-19a-3p miR-19b-3p miR-20a-5p
			miR-20b-5p miR-216b-5p miR-29c-3p
			miR-301a-3p miR-599 miR-93-5p
GS1-358P8.4	Up	Chromosome 16: 68,224,713-68,227,734	miR-106a-5p miR-106b-5p miR-107
			miR-17-5p miR-18a-5p miR-18b-5p
			miR-190b miR-196b-5p miR-20a-5p
			miR-20b-5p miR-216a-5p miR-372-3p
			miR-382-5p miR-497-5p miR-542-3p
			miR-93-5p
RP11-96D1.10	Down	Chromosome 16: 68,224,713-68,227,734	miR-199b-5p miR-202-3p miR-222-3p
			miR-497-5p miR-9-5p

*Down, downregulated in ectopic endometrial samples vs. eutopic endometrial samples; Up, upregulated in ectopic endometrial samples vs. eutopic endometrial samples.*

## Discussion

Endometriosis is a common progressive and recurrent benign disease of the female reproductive tract. In accordance with the definition of endometriosis, ovarian endometriosis is one of the three main types of endometriosis. Endometrial fragments subsequently adhere to an ectopic location and form lesions, because endometriosis tissues are malignant-like, more invasive, and proliferative (Wingfield et al., 1995; Lucidi et al., 2005). Like studies in cancer, it is essential to discover those gene alterations which mediate the development of endometriosis to detect potential biomarkers and treatment targets.

While great efforts have been made to provide the novel insights into the molecular mechanisms of endometriosis in the past years, most studies have focused on elucidating the molecular mechanism that involved in protein-coding genes and miRNAs. Recent years, lncRNA has been demonstrated to play important roles in the biological processes of endometriosis (Panir et al., 2018). CeRNAs including lncRNAs and mRNAs could mutually regulate each other via competing for their shared miRNAs, which are important for physiological and pathological processes of disease. And it has been provided to be an acceptable mechanism of lncRNA function (Tay et al., 2014). However, there was few systematical analysis for lncRNA-mRNA ceRNA crosstalk in endometriosis to reveal those functional lncRNAs.

In the present study, we investigate to detect those functional lncRNAs through constructing lncRNA-mRNA regulatory network based on ceRNA hypothesis. We constructed ovarian endometriosis related ceRNA network combining those differentially expressed lncRNAs, miRNAs and mRNAs. To further explore the hubs in the network, we performed topological feature analysis and finally identified two lncRNAs H19 and GS1-358P8.4 which can be seen as hub regulators. There have many reports about H19 in endometriosis. H19 could reduce the bioavailability of miRNA let-7 and its downstream target IGF1R, to inhibit the proliferation of endometrial stromal cells (Ghazal et al., 2015). Liu S. et al. (2019) found that downregulation of H19 can inhibit ectopic endometrial cell proliferation and invasion by modulating miR-124-3p and ITGB3, while Xu et al. (2019) reported that H19 regulates ACTA2 expression via competition for inhibitory miR-216a-5p binding sites, and that alterations in the estrogen/H19/miR-216a-5p/ACTA2 pathway regulate eutopic endometrial stromal cell invasion and migration. Others also found that H19 overexpression suppressed Th17 cell differentiation and endometrial stromal cells proliferation through the miR-342-3p/IER3 pathway in endometriosis (Liu Z. et al., 2019). Our study may shed further light on the role of H19 in the development of endometriosis.

We also performed KEGG pathway and GO analyses, using the mRNA regulatory targets of H19 and GS1-358P8.4 to investigate the lncRNAs’ functions. We found that the mRNAs targeted by H19 were enriched for genes associated with tight junctions, which have roles in the migration and invasion of endometriotic cells. Based on our findings and existing literature about the role of H19 in endometriosis, we hypothesize that H19 may mediate the process of invasion and migration in endometriosis, although further studies of H19 in endometriosis are still needed. There have been no reports of GS1-358P8.4 having a role in endometriosis, but our KEGG pathway and GO analyses showed that it is associated with transcriptional misregulation in cancer, proteoglycans in cancer, and the Rap1 signaling pathway. As these functions are mainly associated with cancers, we surmise that GS1-358P8.4 may mediate the malignant-like features of endometriotic cells.

As many endometriosis-related genes have already been identified in previous studies and are available on public databases, we also used an RWR algorithm to identify endometriosis-related lncRNAs, using known endometriosis-related genes as seeds. We detected only one lncRNA, RP11-96D1.10, which has the potential to be an important functional lncRNA in endometriosis. It interacts with five miRNAs, of which miR-202-3p was reported to promote the proliferation and metastasis of endometrial cells (Zhang et al., 2015). Using RP11-96D1.10 and its five interacting miRNAs, we were able to discriminate between EC and EU samples. These six RNAs could therefore be used as candidate biomarkers to detect EC tissues. Further studies will need to be performed on the role of RP11-96D1.10 and its potential use as a diagnostic biomarker in endometriosis.

There are, however, some limitations to our study. Firstly, we constructed the endometriosis-related ceRNA network by integrating gene expression and miRNA-target interactions using the hypergeometric test, but our results would be more reliable if a more accurate algorithm were used. Secondly, we performed a bioinformatics-only analysis to detect functional lncRNAs involved in ovarian endometriosis, with results evaluated based on statistical significance and searches of the scientific literature. In the future, we will perform biological experiments to validate these findings.

## Conclusion

In conclusion, our study provided a global view of ceRNA crosstalk between mRNAs and lncRNAs in ovarian endometriosis, and identified three functional lncRNAs, H19, GS1-358P8.4, and RP11-96D1.10 that are strongly associated with the development of endometriosis. The mechanism of action of these lncRNAs in endometriosis still remains to be determined.

## Data Availability Statement

Publicly available datasets were analyzed in this study. This data can be found here: Gene Expression Omnibus (GEO) database (GSE105764 and GSE105765).

## Author Contributions

JB searched the literature, collected the data, and wrote the manuscript. BW analyzed the data and revised the manuscript. TW conceived the project and revised the manuscript. WR designed the project, analyzed the data, and revised the manuscript. All authors contributed to the article and approved the submitted version.

## Conflict of Interest

The authors declare that the research was conducted in the absence of any commercial or financial relationships that could be construed as a potential conflict of interest.

## Footnotes

1 http://www.gencodegenes.org/

2 http://starbase.sysu.edu.cn/

3 http://www.disgenet.org
